# Diagnosis of Complex Regional Pain Syndrome I Following Traumatic Axonal Injury of the Corticospinal Tract in a Patient with Mild Traumatic Brain Injury

**DOI:** 10.3390/diagnostics10020095

**Published:** 2020-02-10

**Authors:** Sung Ho Jang, You Sung Seo

**Affiliations:** Department of Physical Medicine and Rehabilitation, College of Medicine, Yeungnam University, 317-1, Daemyungdong, Namku, Taegu 705-717, Korea

**Keywords:** complex regional pain syndrome, corticospinal tract, traumatic axonal injury, diffusion tensor tractography, mild traumatic brain injury

## Abstract

A 54-year-old male suffered from direct head trauma resulting from a fall while working. At approximately two months after the accident, he began to feel pain (burning sensation) and swelling of the dorsum of the right hand and wrist. He showed the following clinical features among the clinical signs and symptoms of revised diagnostic criteria for complex regional pain syndrome (CRPS): spontaneous pain, mechanical hyperalgesia, vasodilation, skin temperature asymmetries, skin color changes, swelling, motor weakness. No specific lesion was observed on brain MRI taken at ten weeks after onset. Plain X-ray, electromyography, and nerve conduction studies for the right upper extremity detected no abnormality. A three-phase bone scan showed hot uptake in the right wrist in the delayed image. On two-month diffusion tensor tractography, partial tearing of the corticospinal tract (CST) was observed at the subcortical white matter in both hemispheres (much more severe in the left CST). In addition, the fiber number of the right CST was significantly decreased than that of seven normal control subjects. CRPS I of the right hand in this patient appeared to be related to traumatic axonal injury of the left CST following mild traumatic brain injury.

**Figure 1:**
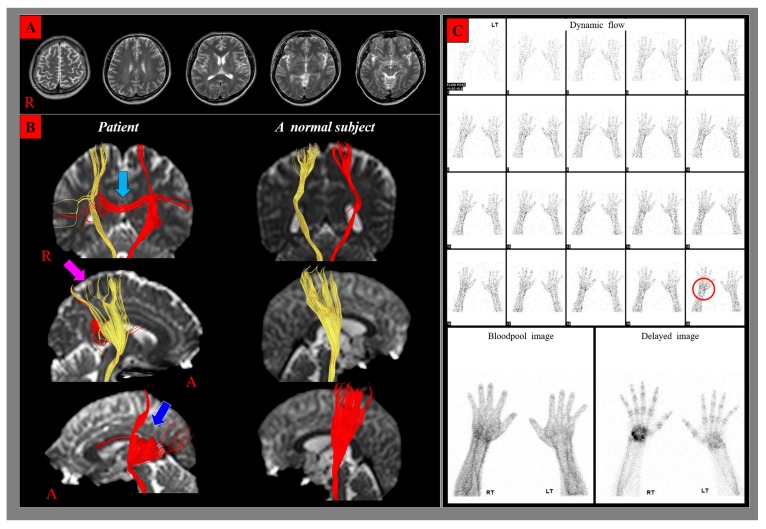
No specific lesion was observed on brain magnetic resonance images (T1-weighted, T2-weighted, and fluid attenuated inversion recovery images) taken at ten weeks after onset (**A**). On two-month diffusion tensor tractography (DTT), partial tearing of the CST was observed at the subcortical white matter in both hemispheres. The partial tearing of the CST was much more severe in the left CST (blue arrow) compared with the right CST (pink arrow) and transcallosal fibers (sky blue arrow) from the left corticospinal tract suggesting the CST injury was also observed (**B**). Plain X-ray, electromyography, and nerve conduction studies for the right upper extremity detected no abnormality. Three-phase bone scan showed that tracer distribution was higher in the right wrist at dynamic flow between the first and last image (red circle), and blood pool image. The delayed image revealed hot uptake in the right wrist and increased tracer uptake in the small joints of the right hand (**C**) [1,2,3]. (R: right, A: anterior, RT: right, LT: left).

A 54-year-old right-handed male suffered from direct head trauma resulting from a fall from a two-meter height while working. He lost consciousness for approximately five minutes and experienced post-traumatic amnesia for approximately 30 min after the accident. His Glasgow Coma Scale score was 15 [4]. Following the accident, the patient developed mild weakness of all four extremities (G^+^ / G^+^), especially right hand (finger flexor: G^−^, finger extensor: G^−^). At approximately two months after the accident, he began to feel pain (burning sensation, visual analogue scale: 7) and swelling of the dorsum of the right hand and wrist. He showed the following clinical features among the clinical signs and symptoms of revised diagnostic criteria for complex regional pain syndrome (CRPS): spontaneous pain, mechanical hyperalgesia, vasodilation, skin temperature asymmetries, skin color changes, swelling, motor weakness [5,6,7]. Diffusion tensor imaging data were acquired two months after the accident using a sensitivity-encoding head coil on a 1.5-T Philips Gyroscan Intera (Hoffman-LaRoche Ltd., Best, the Netherlands) with single-shot echo-planar imaging and navigator echo. For each of the 32 non-collinear diffusion-sensitizing gradients, 67 contiguous slices were acquired parallel to the anterior commissure-posterior commissure line. Imaging parameters were as follows: acquisition matrix = 96 × 96; reconstructed matrix = 128 × 128; field of view = 221 × 221 mm^2^; TR = 10,726 ms; TE = 76 ms; parallel imaging reduction factor (SENSE factor) = 2; EPI factor = 49; b = 1000 s/mm^2^; NEX = 1; and a slice thickness of 2.3 mm with no gap (acquired voxel size 1.25 × 1.25 × 2.5 mm^3^). Fiber tracking used the fiber assignment continuous tracking algorithm implemented within the diffusion tensor imaging task card software (Philips Extended MR Work Space 2.6.3, Philips, Amsterdam, Netherlands). Each diffusion tensor imaging replication was intra-registered to the baseline “b0” images to correct for residual eddy-current image distortions and head motion effect, using a diffusion registration package (Philips Medical Systems). For analysis of the corticospinal tract (CST), fiber tracking used the fiber assignment continuous tracking algorithm implemented within the diffusion tensor imaging task card software (Philips Extended MR Work Space 2.6.3, Philips, Amsterdam, Netherlands). For reconstruction of the CST, the first region of interest was placed on the upper pons (portion of anterior blue color) on the color map with an axial image. The second region of interest was placed on the mid pons (portion of anterior blue color) on the color map with an axial image. The termination criteria used for fiber tracking were fractional anisotropy < 0.1, angle < 27° [8]. On two-month diffusion tensor tractography (DTT), partial tearing of the CST was observed at the subcortical white matter in both hemispheres. The partial tearing of the CST was much more severe in the left CST (blue arrow) compared with the right CST (pink arrow) (**B**). Transcallosal fibers (sky blue arrow) from the left corticospinal tract suggesting the CST injury was also observed (**B**) [9,10]. The fractional anisotropy, apparent diffusion coefficient and fiber number (FN) of the CST were obtained in both hemispheres of the patient and normal control subjects (seven right-handed age- and sex-matched normal control subjects without a history of neurological, physical, or psychiatric illness, mean age 56.5 ± 5.1 years). Statistical analyses were performed using SPSS software (v. 25.0; SPSS, Chicago, IL, USA). We performed analysis using Bayesian statistics for the determination of differences in fractional anisotropy, apparent diffusion coefficient, and FN of the patient and the control subjects [11]. The results of the Bayesian statistical analyses comparing DTT parameters of the patient and control subjects are summarized in Table 1. Significant differences were not observed for the fractional anisotropy, apparent diffusion coefficient, and FN values of the right CST, and the fractional anisotropy and apparent diffusion coefficient value of the left CST between the patient and control subjects (*p* > 0.05). By contrast, the FN value of left CST showed a significant difference compared with that of the control subjects (*p* < 0.05). FN is determined by the included number of voxels in a neural tract, suggesting the total number of fibers of a neural tract [12,13]. Therefore, the decreased FN in the left CST appeared to indicate traumatic axonal injury of this neural tract [14,15].

Brain injury is a precipitating factor of CRPS I [7,16,17,18,19]. The patient’s DTT revealed partial tearing of both CSTs, indicating traumatic axonal injury of the CST [14,15]. Furthermore, the left CST showed a significant decrement of the FN. The FN value indicates the number of voxels included in a neural tract, thereby suggesting the total number of fibers within that tract [20,21,22]. Therefore, the low FN value for the left CST can indicate an injury of the left CST. Considering the results on DTT configuration and parameters, it appeared that the left CST had more severe injury than the right CST. As a result, CRPS I of the right hand in this patient appeared to be related to traumatic axonal injury of the left CST following mild traumatic brain injury. Several studies have reported on CRPS I in patients with traumatic brain injury [16,17]. However, to our best knowledge, this is the first study to report an association of CRPS I with traumatic axonal injury of the CST in a patient with mild traumatic brain injury [14,15,22]. By contrast, a previous study reported a patient with mild traumatic brain injury that was misdiagnosed as CRPS I [23]. The patient was diagnosed based on the clinical features of hyperalgesia with mild edema and motor weakness of both legs without additional supportive evidence from plain radiography of hand and leg, three-phase bone scan, and thermography. Moreover, the patient did not have a history of the distal edema, which is a characteristic feature of acute stage CRPS, and failed to show tropic changes of skin and nails at the chronic CRPS stage. The authors diagnosed the patient as traumatic axonal injury in mild traumatic brain injury based on the DTT evidence that the patient’s clinical features similar to CRPS I were ascribed to traumatic axonal injuries of each neural tracts (hyperalgesia due to the spinothalamic tract injury, and the mild edema and motor weakness of the legs due to the corticospinal and corticoreticulospinal tract injuries) [23].

## Figures and Tables

**Table 1:** Results of Bayesian statistics analyses of diffusion tensor tractography parameters of the corticospinal tract between the patient and control subjects.

		Diffusion Tensor Tractography		
CST		Patient	Control Subjects	*p*-Value
FA	RT	0.47	0.49 ± 0.02	0.21
	LT	0.46	0.47 ± 0.02	0.39
ADC	RT	0.86	0.85 ± 0.03	0.26
	LT	0.84	0.84 ± 0.02	0.29
FN	RT	1654	1711.15 ± 215.18	0.43
	LT	1203	1690.44 ± 194.62	0.01 *

CST: corticospinal tract, FA: fractional anisotropy, ADC: apparent diffusion coefficient, FN: fiber number; * significant differences between the patient and control subjects, *p* < 0.05.
